# Phenotypic and genotypic analysis of benzimidazole resistance in reciprocal genetic crosses of *Haemonchus contortus*

**DOI:** 10.1016/j.ijpddr.2021.11.001

**Published:** 2021-12-01

**Authors:** A.A. Morrison, U. Chaudhry, L. Andrews, L. Melville, S.R. Doyle, N.D. Sargison, D.J. Bartley

**Affiliations:** a Disease Control, Moredun Research Institute, Pentlands Science Park, Bush Loan, Penicuik, Midlothian, EH26 0PZ, United Kingdom; b University of Edinburgh, Royal (Dick) School of Veterinary Studies, Easter Bush Veterinary Centre, Roslin, Midlothian, EH25 9RG, United Kingdom; c Wellcome Sanger Institute, Hinxton, Cambridgshire, CB10 1SA, United Kingdom

**Keywords:** Benzimidazole resistance, Deep amplicon sequencing, Egg hatch test, Reciprocal genetic cross, Pyrosequencing, Single nucleotide polymorphisms

## Abstract

*Haemonchus contortus* is arguably one of the most economically important and ubiquitous parasites of livestock globally and commonly involved in cases of anthelmintic resistance. Here, we performed reciprocal genetic crosses using susceptible (MHco3(ISE)) and multiple anthelmintic resistant (MHco18(UGA2004)) *H. contortus* isolates. Resultant admixed populations were designated MHco3/18 or MHco18/3, where the lead isolate reflects the origin of the females. Three independent filial generations were generated for each cross, which were subjected to bioassays, molecular approaches and population genetic analyses to investigate the phenotypic and genotypic inheritance of benzimidazole (BZ) resistance at each stage. A panel of microsatellite markers confirmed the success of the genetic cross as markers from both parents were seen in the F_1_ crosses. Egg hatch tests revealed a stark difference between the two F_1_ crosses with ED_50_ estimates for MHco18/3 being 9 times greater than those for MHco3/18. Resistance factors based on ED_50_ estimates ranged from 6 to 57 fold in the filial progeny compared to MHco3(ISE) parents. Molecular analysis of the F167Y and F200Y SNP markers associated with BZ resistance were analysed by pyrosequencing and MiSeq deep amplicon sequencing, which showed that MHco3/18.F_1_ and MHco18/3.F_1_ both had similar frequencies of the F200Y resistant allele (45.3% and 44.3%, respectively), whereas for F167Y, MHco18/3.F_1_ had a two-fold greater frequency of the resistant-allele compared to MHco3/18.F_1_ (18.2% and 8.8%, respectively). Comparison between pyrosequencing and MiSeq amplicon sequencing revealed that the allele frequencies derived from both methods were concordant at codon 200 (*r*_*c*_ = 0.97), but were less comparable for codon 167 (*r*_*c*_ = 0.55). The use of controlled reciprocal genetic crosses have revealed a potential difference in BZ resistance phenotype dependent on whether the resistant allele is paternally or maternally inherited. These findings provide new insight and prompt further investigation into the inheritance of BZ resistance in *H. contortus*.

## Highlights

• Reciprocal cross used to investigate benzimidazole (BZ) resistance.
• Phenotypic and genotypic tools combined for analysis.
• Inheritance of BZ resistance influenced by maternal &/or cytoplasmic mechanisms.
• Double homozygous resistant genotypes at F167Y and F200Y detected on β−tubulin gene.

## Graphical abstract

**Figure:**
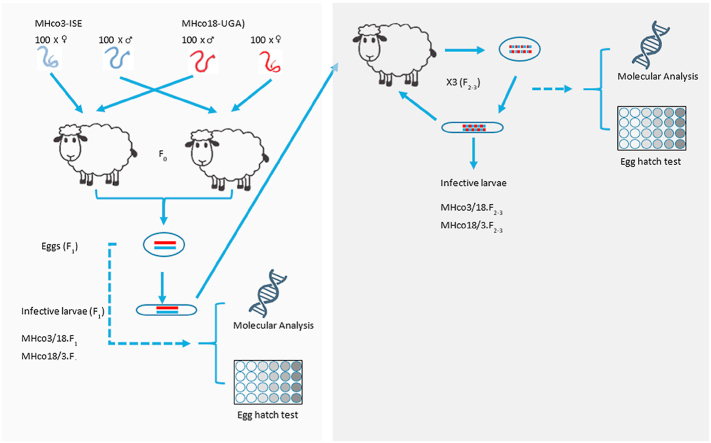

## 1 Introduction

Resistance to each of the major broad-spectrum classes of anthelmintics has emerged rapidly in parasitic nematodes of small ruminants and is now widespread in several species, including *Teladorsagia circumcincta* and *Haemonchus contortus* (Kaplan, 2004). Resistance to the benzimidazole (BZ) class of anthelmintics was first reported in *H. contortus* in 1964 (Drudge et al., 1964) and is now prevalent worldwide (Fitzpatrick, 2013).

Understanding the evolution and inheritance of anthelmintic resistance has been a global research focus for many years. Developments in the field of BZ resistance are the most advanced for any of the anthelmintic classes, although questions relating to the inheritance of resistance genes still exist. A number of non-synonymous single nucleotide polymorphisms (SNPs) on the β-tubulin isotype 1 gene have been associated with BZ resistance in several helminth species, and include a phenylalanine to tyrosine substitution at codon 200 (F200Y) (Kwa et al., 1994), phenylalanine to tyrosine (F167Y) (Ghisi et al., 2007) or phenylalanine to histidine at codon 167 (F167H) (Prichard, 2001; Silvestre and Cabaret, 2002), and glutamic acid to alanine at codon 198 (E198A) (Silvestre and Cabaret, 2002). Most recently, a change at codon 198 was reported in *H. contortus* and *T. circumincta* where glutamic acid switched to leucine (E198L) and was found to be independent of F167Y and F200Y (Mohammedsalih et al. 2020; Martínez-Valladares et al., 2020). Similarly, other changes were observed in *H. contortus* where gulatmic acid changed to either valine, lysine or isoleucine (E198L/E198V/E198K/E198I) (Mohammedsalih et al., 2020). In *H. contortus*, the β-tubulin isotype 1 gene (HCON_00005260) is autosomal and is located on chromosome 1 at position 7027492-7031447; in globally distributed populations, this genomic locus remains highly differentiated as a result of longterm and widespread use of BZ drugs that have selected for resistance (Doyle et al., 2020). The presence of the β-tubulin SNPs appears to be well correlated with phenotypic expression of BZ resistance (von Samson-Himmelstjerna et al., 2007), however, the relative impact of the different SNPs towards the resistance phenotype and interactions between them remain unclear (Kotze et al., 2014).

Various worm mating protocols have been used to explore the genetics and inheritance of anthelmintic resistance, often with conflicting findings. BZ resistance in *H. contortus* was reported to be semi-dominant (Le Jambre et al., 1979), and that a matroclinous influence on the *in vitro* expression of BZ resistance was observed, putatively due to the maternal contribution to egg cytoplasm and shell formation. They also suggested extra-chromosomal inheritance of some traits. Martin et al. (1988) also identified a strong maternal effect in the inheritance of resistance to the BZ drug, thiabendazole (TBZ) in *Trichostrongylus colubriformis*. However, Sangster et al. (1998) found, using different isolates, that resistance was an incompletely recessive, autosomal trait suggesting that more than one gene was involved in resistance (Le Jambre et al., 1979; Herlich et al., 1981), and found little to no evidence for maternal effects on inheritance in F_1_ generations of *H. contortus*. However, these previous reciprocal F_2_ genetic crosses have not been pursued with molecular analysis of markers associated with drug resistance.

Here, we describe the phenotypic and genotypic analysis of BZ resistance in reciprocal genetic crosses of the susceptible (MHco3(ISE)) and multiple anthelmintic class resistant (MHco18(UGA2004)) *H. contortus* isolates. Using combined applied *in vivo* and *in vitro* techniques to follow phenotypic traits of the crosses together with genetic analyses using microsatellite makers, pyrosequencing and deep amplicon sequencing, we sought to investigate: (i) the influence of maternal versus paternal inheritance of resistant alleles on the phenotypic expression of BZ resistance; and (ii) how the two most common BZ resistance associated SNPs (F167Y and F200Y) are inherited following reciprocal genetic crosses between the resistant MHco18(UGA2004) and susceptible MHco3(ISE) isolate.

## 2 Materials and methods

### 2.1 *H. contortus* isolates

Two parental isolates were selected to undertake the initial cross. MHco3(ISE) is an anthelmintic drug susceptible *H. contortus* isolate that was inbred over 15 generations of half sibling matings and has been maintained in the lab at Moredun Research Institute, UK since 2004 (Roos et al., 1990, 2004). MHco18(UGA2004) is a BZ, levamisole and ivermectin resistant *H. contortus* isolate that was recovered from sheep in 2004 and maintained in the lab at University of Georgia, USA (Williamson et al., 2011).

### 2.2 Setup of reciprocal genetic crosses

Reciprocal crosses are designed to examine the role that each parental sex plays in the inheritance of traits. Using the reciprocal F2 genetic cross approach outlined below meant that any sex-linked traits, either in phenotype or genotype, associated with BZ resistance could be identified. Reciprocal genetic crosses between MHco3(ISE) and MHco18(UGA2004) were carried out as previously described by Doyle et al., (2019). However, in this case, both isolates were used as the dam of a cross to allow for comparison and any sex-linked traits to be highlighted.

**Fig. 1:**
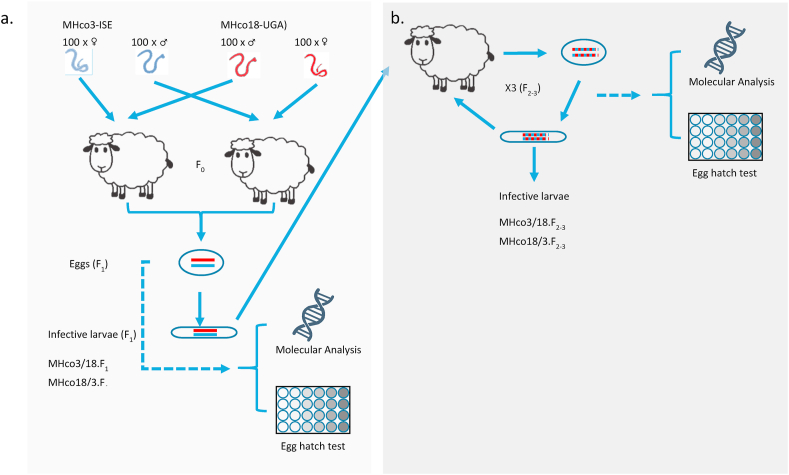
Outline of the reciprocal crosses, filial crosses and *in vivo* passage. a. One hundred L_4_/immature female adults of a multi resistant population MHco18(UGA2004) (“resistant” haplotypes depicted as red lines) were crossed with 100 L4/immature male adults of a susceptible population MHco3(ISE) (“susceptible” haplotypes as blue lines) to generate heterozygous F1 progeny. A reciprocal cross was also initiated with 100 L4/immature female MHco3(ISE) which were crossed with 100 L4/immature male MHco18(UGA2004). Eggs were collected and cultured to generate infective larvae for subsequent infections from each cross b. Three more filial generations were derived by infecting parasite naïve lambs with 5000 infective larvae that were derived from the previous infection. (For interpretation of the references to colour in this figure legend, the reader is referred to the Web version of this article.)

One hundred MHco3(ISE) females and 100 MHco18(UGA2004) males were surgically transferred to one parasite-naive male recipient lamb, and 100 MHco18(UGA2004) females and 100 MHco3(ISE) males were surgically transferred to another parasite-naive male lamb to achieve reciprocal genetic crosses (Fig. 1). The genetic crosses were designated MHco3/18 and MHco18/3, representing female/male parents. Faeces were collected from recipients seven days post-surgery to collect eggs and culture L_3_, designated F_1_. 5000 F_1_ L_3_ from the two different crosses were administered *per os* to parasite naïve male lambs to generate F_2_ populations. Faecal material was collected, from which parasites were cultured to infect two further worm-naive male lambs to generate a F_3_ population.

The cross was undertaken with a view to the future, as other markers may be developed for resistance to other drug families, and materials were archived accordingly for further investigation. At all stages in the process parasitic material was preserved. Adults and L_3_ from each generation were snap frozen in liquid nitrogen and stored at −70 °C for subsequent molecular analysis. Additionally, sufficient L_3_ from each generation were stored in liquid nitrogen so they could be resurrected for future experimental infections.

### 2.3 Faecal worm egg counts

A modification of the salt flotation faecal worm egg count (FWEC) method described by Jackson (1974) was used, with a sensitivity of up to one egg per gram. All FWECs presented are from single male lambs and, therefore, statistical analysis is not possible other than to calculate the basic range, arithmetic mean, and cumulative egg output for each animal. All filial generation FWECs were performed weekly from 18 to 21 days post infection.

### 2.4 Coproculture

Faecal material was collected and cultured from lambs throughout the study to generate infective larvae (L_3_) for subsequent infections using the protocols as outlined previously (Coop et al., 1982). The *H. contortus* larvae were stored at 8-10 °C, and used within 6 months of collection.

### 2.5 Benzimiadazole efficacy

To confirm the BZ drug sensitivity profile and to generate material for analysis, the MHco3/18.F_3_ and MHco18/3.F_3_ generations were treated at the manufacturer's recommended dose rate with fenbendazole (FBZ; Panacur, MSD Animal Health; 5 mg/kg bodyweight). The FBZ treated genetic cross populations were subsequently referred to as MHco3/18.F_3_.BZ and MHco18/3.F_3_.BZ. Lambs (n = 1 for each genetic cross). Efficacies of drug treatment were calculated using the following faecal egg count reduction test (FECRT) calculation (Kochapakdee et al., 1995):

Percentage efficacy = (1 − [ FWEC_Day10_ / FWEC_Day0_ ]) x 100

where FWEC_Day0_ and FWEC_Day10_ are the FWECs of the lambs on day of FBZ administration and 10 days later, respectively.

### 2.6 Egg hatch test and determination of dominance

Egg hatch tests were performed for each filial generation and for the F_3_ population treated with a BZ drug on day 53 post infection.

The egg hatch test was conducted as previously described (von Samson-Himmelstjerna et al., 2009a). The final thiabendazole (TBZ) drug concentrations examined ranged between 0 and 5 μg/ml TBZ. Each drug concentration was made up independently from a stock solution of 1 mg/ml TBZ in dimethyl sulfoxide (DMSO) and set up in triplicate for each test. Each egg hatch test was set up three times during the course of each patent infection. An additional test was also carried out post-FBZ treatment of the F_3_ generation. Eggs/first stage larvae within each test well were fixed in ethanol (70% v/v) prior to analysis and to preserve material for future molecular studies. Egg death 50 (ED_50_) estimates were calculated for each generation of genetic cross. Probit analysis (Minitab 16 statistical software, Minitab LLC, USA) was used to calculate the ED_50_ estimates and standard error of the mean (SEM) for the egg hatch tests. Resistance factors were calculated by dividing the ED_50_ estimates with the susceptible parent MHco3(ISE) ED_50_.

Degrees of dominance (D) based on the log transformed egg hatch ED_50_ estimates were calculated using the previously published Falconer's equation (Stone, 1968).

$$\text{D}=\left(2ED_{50f}-ED_{50r}-ED_{50s}\right)\;÷\;\left(ED_{50r}-ED_{50s}\right)$$

In this equation, $ED_{50f},\;ED_{50r}\;\text{and}\;ED_{50s}$ represent ED_50_ estimates of the F_1_ crosses, MHco18(UGA2004), and MHco3(ISE), respectively. D = 1 would be indicative of complete dominance; 0 < D < 1 of incomplete dominance; −1 < D < 0 of incomplete recessivity; and D = −1 of complete recessivity.

### 2.7 Single nucleotide polymorphism (SNP) and allele quantification

#### 2.7.1 Pyrosequencing

Individual infective larvae (n = 88) from each filial generation of the reciprocal cross and the BZ treated F_3_ populations were picked into separate wells of a 96-well plate (Axygen, USA) containing 25 μl of worm lysis buffer (Kwa et al., 1995). Proteinase K (Promega, UK) was added to each well to create a final concentration of 100 μg/ml enzyme. Eight non-template control wells per plate were included. Plates were placed at −80 °C for 4 h before incubation at 56 °C overnight to allow for lysis of the parasite material. Lysates were heated to 90 °C for 30 min to deactivate the proteinase K. Lysates were stored at −20 °C prior to analysis.

The primers used to analyse the F167Y and F200Y SNPs of the β-tubulin isotype 1 gene have been previously described (von Samson-Himmelstjerna et al., 2009b). The codon 198 SNPs (E198A, E198L, E198V, E198K or E198I) were not examined as they are not present in the MHco18(UGA2004) and MHco3(ISE) isolates, confirmed by whole-genome sequencing (Doyle et al., 2021). For pyrosequencing PCR, 4 μl of gDNA crude lysate was included in each 50 μl reaction. NovaTaq™ Hot start master mix (Merck, UK) was used for the PCR step containing 0.2 μM Forward primer, 0.185 μM Reverse biotinylated primer, 1.5 mM MgCl_2_, 25 μl 2 × buffer and made up to 50 μl using DNA/RNA-free water.

Following a 15 min 95 °C polymerase activation step, amplification was performed using 45 cycles of 94 °C for 30 s, 53 °C for 30 s and 72 °C for 30 s, followed by a final extension step at 72 °C for 10 min. To confirm amplification, 10 μl of each PCR reaction was examined by gel electrophoresis on 2% agarose gels stained with gel red (Biotium, California, USA). The remaining 40 μl of the reaction was analysed by pyrosequencing on a PyroMark ID instrument (Qiagen, Germany) following the manufacturer's protocol.

#### 2.7.2 Genotyping based on pyrosequencing results

SNP's at codons 167 and 200 were noted for each L_3_ (TT – homozygous susceptible - SS; AT heterozygote – SR; and AA – homozygous resistant - RR). Resistant (R) and susceptible (S) allele frequencies were counted and expressed as a percentage for each generation. Genotype combinations for both SNPs were counted and expressed as percentages. Pairwise comparison of genotypes was carried out using Shannon Diversity Indices where outputs are G test values and chi square probabilities. The populations were also checked for Hardy Weinberg Equilibrium and Fixation index (inbreeding coefficient) using the GenAlEx plug-in in Microsoft Excel (Microsoft corporation, USA) version 6.5 (Peakall and Smouse, 2012).

### 2.8 Deep amplicon sequencing

Pooled larvae from each filial generation derived from the reciprocal cross and the MHco3/18 BZ treated F_3_ population were used to create crude lysates for deep amplicon sequencing of a 328 bp fragment of the *H. contortus* β-tubulin isotype 1 gene that spanned the codons F200Y (TTC-TAC), E198A (GAA-GCA), F167Y (TTC-TAC) and the intervening intron. The modified primer sets, adapter/barcoded PCR amplification conditions and AMPure XP Magnetic Beads (1X) (Beckman Coulter, Inc.) purification were previously described by Ali et al. (2018). Ten μl of each barcoded PCR product was combined to make a pooled library. Pooled libraries were run on agarose gel electrophoresis to separate PCR products. The desired β-tubulin isotype 1 PCR amplicon was excised from the gel from which DNA was isolated by gel extraction purification (QIAquick Gel Extraction Kit, Qiagen, Germany). The eluted 20 μl DNA was then purified using AMPure XP Magnetic Beads (1X) (Beckman Coulter, Inc.) to produce a single purified DNA pool library. The library was first measured with a KAPA qPCR library quantification kit (KAPA Biosystems, USA) and then run on an Illumina MiSeq sequencer using a 600-cycle pair-end reagent kit (MiSeq Reagent Kits v2, MS-103-2003) at a concentration of 15 nM with the addition of 15% PhiX Control v3 (Illumina, FC-11-2003).

A post MiSeq analysis separates all the sequence by sample via the recognised barcoded indices and generates the FASTQ files. The data analysis was performed with a bespoke pipeline using Mothur v1.39.5 software (Schloss et al., 2009) with modifications in the standard operating procedures of Illumina MiSeq in the previously described Command Prompt pipeline (Ali et al., 2018; Sargison et al., 2019b). Briefly, the ‘make.contigs’ command was run on raw paired-end reads from each sample to combine the two sets of reads. The command extracted sequence and quality scores from the FASTQ files, creating the complement of the reverse and forward reads, and then joined the read pairs into contigs. After removing long or ambiguous sequence reads (>328 bp) using the ‘screen.seqs’ command, the data was aligned with the *H. contortus* β-tubulin isotype 1 reference sequence library using the ‘align.seqs’ command. The sequences that did not match with the *H. contortus* β-tubulin isotype 1 reference sequence library were removed and the ‘summary.seqs’ command was used to summarise the 328 bp sequence reads of the *H. contortus* β-tubulin isotype 1 locus. The sequence reads were further run on the ‘screen.seqs’ command to generate the *H. contortus* β-tubulin isotype 1 FASTQ file. Once the sequence reads were classified as β-tubulin isotype 1, a count list of the consensus sequences of each sample was created using the ‘unique.seqs’ command. The count list was further used to create FASTQ files (Mendeley database at https://doi.org/10.17632/57n7p8gxh2.1) of the consensus sequences of each sample using the ‘split.abund’ command to sort data into groups of rare and abundant based on the cutoff value (1000 reads), followed by the ‘split.groups’ command. Consensus sequences for *H. contortus* β-tubulin isotype 1 were generated from the count list using Geneious Prime 2020.1 software (Kearse et al., 2012). These consensus sequences were used for the calculation of the relative allele frequencies of β-tubulin isotype 1 resistance-associated mutations. To achieve this, *H. contortus* β-tubulin isotype 1 were first assigned to susceptible and the relevant resistance mutations based on known SNPs at codons F200Y (TTC-TAC), E198A (GAA-GCA), F167Y (TTC-TAC), followed by dividing the number of sequences reads of each sample that contained the mutation by the total number of reads (R Core Team, 2014).

Genotyping results obtained from the analysis of individual larvae by pyrosequencing and pooled parasite material using deep amplicon sequencing were compared by Lin's Concordance Correlation Coefficient, calculated using the epiR program in R (version 3.6.3).

### 2.9 Microsatellite genotyping of the parental isolates and genetic crossing progeny

Thirty individual larvae of each population including the parental isolate, derived from the initial donor lamb infections described in section 2.2 [MHco3(ISE), MHco18(UGA2004)] and three of the genetic crossing progeny [MHco3/18.F_1_, MHco18/3.F_1_ and MHco3/18.F_3_(BZ)] were analysed. Individual larvae were added into a single 0.2 μl tube containing 20 μl of 10 mg/ml proteinase K (New England Biolabs) and Lysis Reagent (Viagen) (Redman et al., 2008; Chaudhry et al., 2015).

One μl of neat individual worm lysate was used as PCR template and identical dilutions of lysate buffer, made in parallel, were used as negative controls. A panel of six microsatellites (Hcms8a20, Hcms22c03 (Redman et al., 2008); Hcms25, Hcms33 (Otsen et al., 2000); Hcms22193 and Hcms53265 (Redman et al., 2015)) was selected to include potentially useful markers across the genome of *H. contortu*s. The forward primer of each microsatellite primer pair was 5′-labelled with a fluorescent dye (IDT, UK) and the GeneScan ROX 400 internal size standard was used on the ABI Prism 3100 genetic analyser (Applied Biosystems, UK). Individual chromatograms were analysed using Gene Mapper software version 4.0 (Applied Biosystems, UK) to accurately size the amplicons and determine genotypes. Fixation index (pairwise F_ST_) values were calculated from the multi-locus microsatellite genotype data by random permutation in Arlequin 3.11 (Excoffier et al., 2005). Principal coordinate analysis (PCA) was performed using GenAlEx version 6.5 preserving individual worm genotypes (Peakall and Smouse, 2012). A summary of primer sequences, allele ranges and PCR conditions for each marker as used in our hands is given in Supplementary Table S1.

## 3 Ethics statement

All experimental procedures described in this manuscript were examined and approved by the Moredun Research Institute Animal Welfare and Ethical Review Board (E30/14, E47/17 & E27/19) and were conducted under approved British Home Office licenses in accordance with the Animals (Scientific Procedures) Act of 1986.

## 4 Results

### 4.1 Faecal worm egg counts

FWECs from all stages of the crosses are illustrated in Fig. 4 and separated into three panels a, b and c.

The FWECs of both the MHco3/18.F_1_ and MHco18/3.F_1_ recipient lambs were zero eggs per gram (epg) on day 18 post infection (4 days post-surgery) and 306 epg (for both lambs) on day 28 (10 days post-surgery). The FWECs of the MHco3/18.F_1_ and MHco18/3.F_1_ recipient lambs are highlighted in Fig. 4b as this data is lost when looking at the overall picture in Fig. 4a due to their egg counts being lower compared to the other filial generations. The lambs producing the F_2_ populations showed an upward trend in FWEC with a peak in both crosses at day 47 post infection (week 6). Each of the MHco18/3 filial generations maintained a higher FWEC compared to the equivalent MHco3/18 generations (between 19 and 247 days post infection).

The F_3_ populations pre-treatment (day 21–46 post infection) had a similar trend where MHco18/3.F_3_ peaked at day 35 with 8028 epg, 1.5 times higher than MHco3/18.F_3_ FWEC at that timepoint (Fig. 4a). Post FBZ treatment, the FWECs of the lambs infected with both reciprocal cross F_3_ remained positive (Fig. 4c). FBZ treatment showed 77% efficacy against the MHco3/18.F_3_ at 10 days post treatment, whereas the equivalent efficacy against the MHco18/3.F_3_ was 87%.

The cumulative FWEC output was greater in MHco18/3 compared to the MHco3/18 filial populations. The difference between the parental isolates cumulative FWEC was 1.1 where MHco3(ISE) had 423,011 and MHco18(UGA2004) had 472,644. In the F_1_ population MHco18/3 cumulative FWEC was 1.4 times greater than MHco3/18.F_1_ (5232 and 3879, respectively). In the F_2_ population, the cumulative FWEC of MHco18/3.F_2_ was 3.3 times greater than MHco3/18.F_2_ (35,500 and 10,665, respectively). Lastly, in the F_3_ population, the cumulative FWEC of MHco18/3.F_3_ was 1.5 times greater compared to MHco3.18.F_3_ (156,287 and 106,627, respectively).

### 4.2 Egg hatch test

The parental isolate's ED_50_ estimates were 0.026 μg/ml TBZ for MHco3(ISE) and >5 μg/ml TBZ for MHco18(UGA2004). A precise ED_50_ estimate could not be calculated for MHco18(UGA2004) as more than 50% of the eggs hatched at all drug concentrations tested even at the highest concentration tested (Table 2). Generally, the MHco18/3 genetic cross filial generations had higher ED_50_ estimates (with the exception of the F_3_ untreated population) than their reciprocal counterparts, indicative of a higher level of the resistance phenotype. The greatest difference observed between the ED_50_ estimates was between the F_1_ populations where MHco18/3.F_1_ had a 9.8-fold greater ED_50_ estimate compared to the MHco3/18.F_1_. This was reduced to being a 1.2-fold difference in the F_2_ populations. Resistance based on ED_50_ estimates ranged from 6- to 57-fold higher compared to the susceptible MHco3(ISE) parental isolate (Table 2).

**Table 1:** Pairwise F_ST_ values based on genotyping 30 individual worms from parental isolates, F_1_ progeny [MHco18/3.F_1_, MHco3/18.F_1_] and drug selected F_3_ progeny [MHco3/18.F_3_(BZ)] with six microsatellite markers. Pairwise comparisons with statistically significant (p < 0.001).

	MHco3 (ISE)	MHco18 (UGA2004)	MHco3/18.F_1_
MHco18 (UGA2004)	0.2022	–	–
MHco18/3.F_1_	0.0731	0.0823	–
MHco3/18.F_1_	0.0780	0.0770	–
MHco3/18.F_3_.BZ	0.0686	0.0542	0.0372

**Table 2:** The ED_50_ estimates (μg/ml TBZ) along with standard error of the mean (SEM) for each of the reciprocal genetic crosses and filial generations as calculated using probit analysis. The resistance factors are calculated against the MHco3 (ISE) susceptible parent.

Population	Designation	ED_50_ Estimate ± SEM (TBZ μg/ml)	Resistance Factor
Parent	MHco3 (ISE)	0.026 ± 0.001	1
Parent	MHco18 (UGA2004)	>5	>192
F_1_	MHco3/18.F_1_	0.150 ± 0.004	6
F_2_	MHco3/18.F_2_	0.185 ± 0.002	7
F_3_	MHco3/18.F_3_	0.182 ± 0.007	7
F_3_ BZ	MHco3/18.F_3_.BZ	0.494 ± 0.020	19
F_1_	MHco18/3.F_1_	1.470 ± 0.160	57
F_2_	MHco18/3.F_2_	0.229 ± 0.003	9
F_3_	MHco18/3.F_3_	0.177 ± 0.009	7
F_3_ BZ	MHco18/3.F_3_.BZ	0.850 ± 0.030	33

#### 4.2.1 Degrees of dominance

Degrees of dominance estimates from the two reciprocal crosses F_1_ were not in agreement, where MHco3/18 D = −0.389 suggested incomplete recessivity whereas MHco18/3 D = 0.416 suggested incomplete dominance.

### 4.3 Microsatellites

**Fig. 2:**
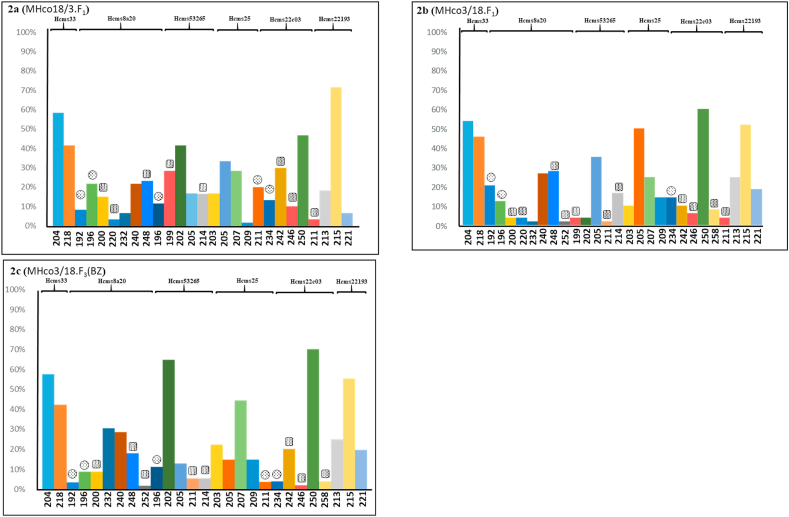
Alleles present (different colour shades) in three crossing progeny [MHco18/3.F_1_, and MHco3/18.F_1,_ MHco3/18.F_3_(BZ)] using six microsatellite markers. Panel a–c: Individual worm genotyping has been performed based on 30 individual L_3_ stage larvae of MHco18/3.F_1_, MHco3/18.F_1_ and MHco3/18.F_3_(BZ) respectively. In each panel, the dotted square icons represents the alleles unique to MHco18(UGA2004) and dotted circles represent those unique to MHco3(ISE) parental isolates, and the unmarked columns refer to alleles found in both parental isolates. X-axis represent the frequency of the alleles, Y-axis represents the bases pair of the alleles in each markers and name of the markers were shown on the top of the figure. (For interpretation of the references to colour in this figure legend, the reader is referred to the Web version of this article.)

The reciprocal genetic crosses using the susceptible MHco3(ISE) and BZ resistant MHco18(UGA2004) *H. contortus* isolates were validated using a panel of six microsatellite markers. The presence or absence of the microsatellite marker alleles allowed the genetic crosses to be monitored, and provided confirmation that they were progressing as expected. Thirty individual L_3_ from each parental isolate, F_1_ progeny and drug selected F_3_ progeny were genotyped. A total of 16 different isolate specific alleles were identified from three out of the six microsatellite markers including five alleles that were present in MHco3(ISE), but absent in MHco18(UGA2004), and 11 alleles that were present in MHco18(UGA2004), but absent in MHco3(ISE) (Supplementary Fig. S1 a & b). The MHco18/3.F_1_ progeny carried all five alleles derived from the MHco3(ISE) parental isolate and eight from MHco18(UGA2004). Three alleles [252 (Hcms8a20), 211 (Hcms53265) and 258 (Hcms22c03)] from the MHco18(UGA2004) parental isolate were absent in the MHco18/3 F_1_ progeny (Fig. 2a). In contrast, MHco3/18.F_1_ progeny carried all 11 alleles derived from the MHco18(UGA2004) parent and two out of five alleles [196 (Hcms53265) and 211 (Hcms25)] from the MHco3(ISE) parental isolate (Fig. 2b). Five MHco3(ISE) specific alleles and two out of 11 alleles [220 (Hcms8a20) and 199 (Hcms53265)] of the MHco18(UGA2004) parental isolates were retained in the MHco3/18.F_3_(BZ) progeny (Fig. 2c).

**Fig. 3:**
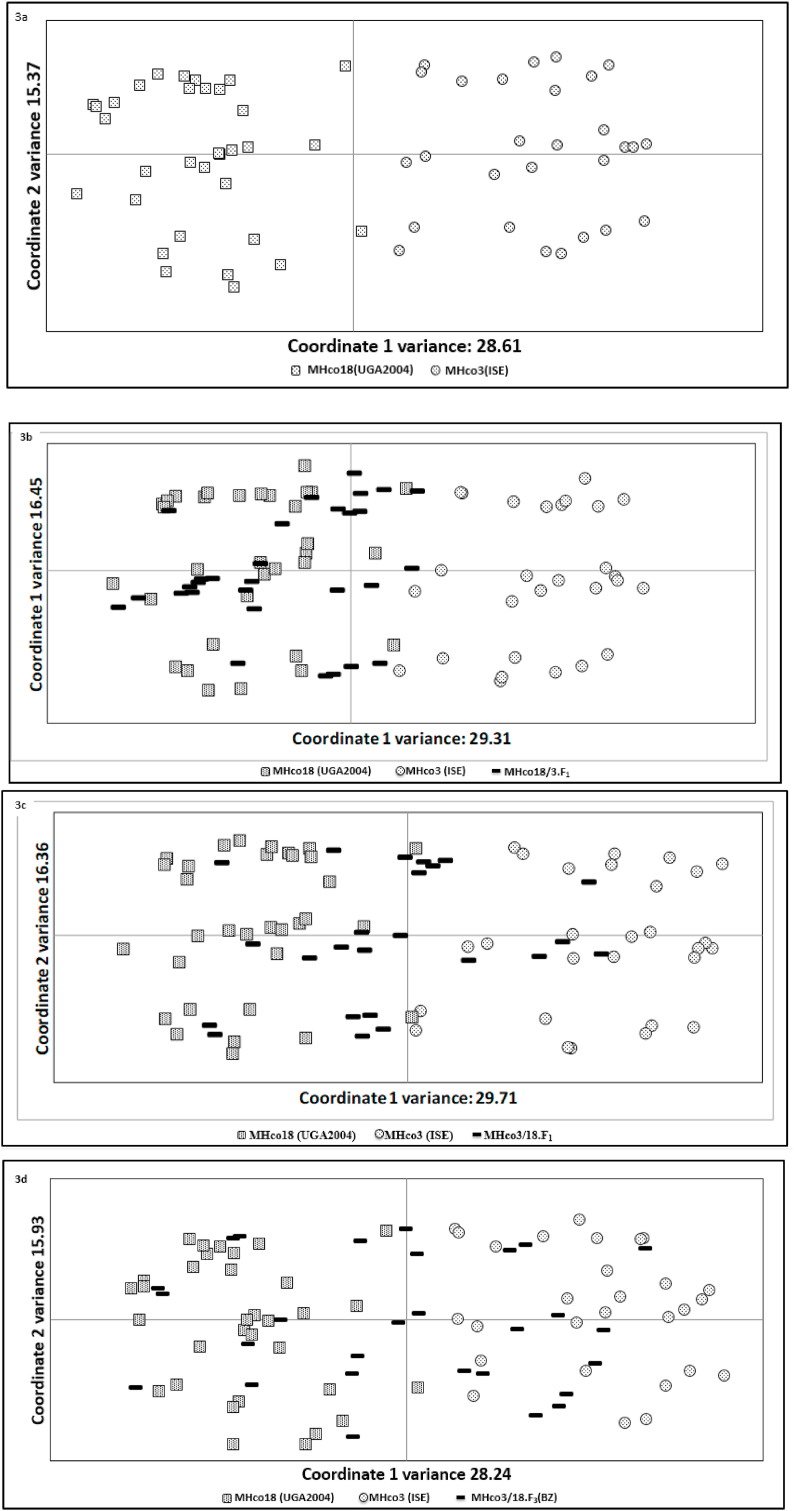
Principle coordinate analysis of microsatellite markers for MHco3(ISE) & MHco18(UGA2004) parental isolates and progeny [MHco18/3.F_1_, MHco3/18.F_1_, MHco3/18.F_3_(BZ)] as shown in panel a–d respectively. Panel a shows the PCA plot comparing the microsatellite markers for the parental isolates. Panels b–d shows the PCA plot comparing the parental isolates with the respective genetic cross filial generation added shown in the black bar. In each panel the square represents MHco18(UGA2004) alleles, the circle represents MHco3(ISE) alleles and black bar represents the alleles which come from the genetic cross: MHco18/3.F_1_ (b), MHco3/18.F_1_ (c) and MHco3/18._F3_(BZ) (d) generations.

Genotyping of the MHco3(ISE) and MHco18(UGA2004) parental isolates using microsatellite markers confirmed a high level of genetic differentiation between MHco3(ISE) and MHco18(UGA2004). The principal coordinate analysis (PCA) of MHco3(ISE) and MHco18(UGA2004) microsatellite data revealed two separate clusters in the plot (Fig. 3a). This genetic differentiation was reflected by the high pairwise F_ST_ estimates calculated between both isolates (F_ST_ 0.2022) (Table 1).

**Fig. 4:**
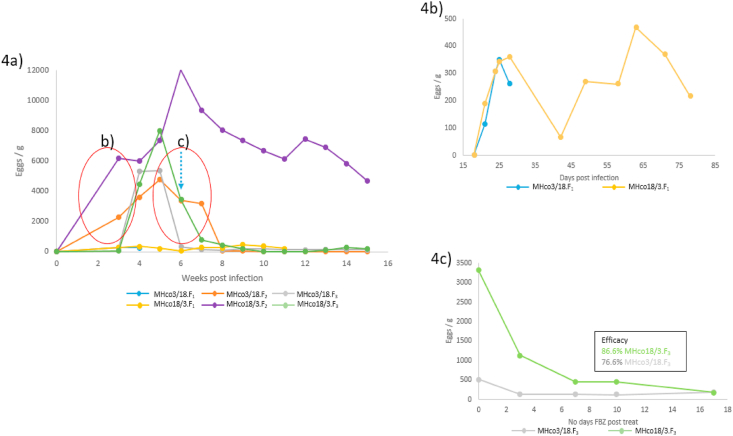
Shows Faecal worm egg counts (eggs/g) of the individual donor animals used in this study split over 3 panels a–c. a. Shows an overview of all the donor animals used in this study (original recipients – F_1_; F_1_ passaged –F_2_; F_2_ passaged – F_3_). Where the blue line represents MHco3/18.F_1_; the yellow line represents MHco18/3.F_1_; the orange line represents MHco3/18.F_2_; the purple line represents MHco18/3.F_2_; the grey line represents MHco3/18.F_3_ and the green line represents MHco18/3.F_3_. The red circles highlight the area of the overview graph being shown in greater detail in panel b & c. The blue dashed arrow depicts when the F_3_ generation was treated with fenbendazole (FBZ). b. Faecal egg counts of the recipient donors used in the original reciprocal crosses. c. Faecal egg count of the individual donors infected with each genetic cross of the F_2_ generation after treatment with FBZ. The text box shows the efficacy of FBZ at 10 days post treatment. (For interpretation of the references to colour in this figure legend, the reader is referred to the Web version of this article.)

The F_1_ generation were, however, much more closely related to each of the MHco3(ISE) and MHco18(UGA2004) parental isolates; in the PCA MHco18/3.F_1_, MHco3/18.F_1_ and MHco3/18.F_3_(BZ) progeny showed clustering of alleles with MHco3(ISE) or MHco18(UGA2004) parental isolates (Fig. 3b, c and d), and were less distinct by measures of F_ST_ [ MHco18/3.F_1_ (F_ST_ = 0.0731 and F_ST_ = 0.0823) and MHco3/18.F_1_ (F_ST_ = 0.0780 and F_ST_ = 0.0770), respectively]. A similar pattern was seen in the comparison MHco3/18.F_3_(BZ) and MHco3(ISE) (F_ST_ = 0.0686) and MHco18(UGA2004) (F_ST_ = 0.0542) parental isolates. Genetic differentiation was lower between the reciprocal MHco3/18.F_1_ and MHco3/18.F_3_(BZ) progenies (F_ST_ = 0.0372) (Table 1).

### 4.4 Pyrosequencing and deep amplicon sequencing

#### 4.4.1 Genotype variability

We determined the frequency of all possible genotype combinations between the F167Y and F200Y loci using pyrosequecing of individual larvae from each stage of both crosses. In total, 9 different genotype combinations were possible (Table 3). MHco18(UGA2004) had a variety of β-tubulin codon 167 and 200 SNP genotype combinations present, with six out of the possible nine combinations detected; it contained no double susceptible genotypes, and a low level of double homozygous resistant genotypes (3.53%) at both SNPs (Table 3). The MHco3(ISE) parental population had 100% homozygous susceptible genotypes at both SNPs. Both reciprocal F_1_ genetic crosses had the same three codon 167-200 genotype combinations (SS-RS, RS-SS and RS-RS). MHco18/3.F_1_ appeared to have more heterozygotes at F167Y compared to MHco3/18.F_1_ (Table 3). In the F_2_ generations, the genotype combinations increased from three to six for MHco3/18.F_2_ and seven for MHco18/3.F_2_ where MHco3/18.F_2_ did not have any genotypes with homozygous resistant F167Y present. In the F_3_ generations pre treatment, both genetic crosses had seven genotype combinations. For MHco3/18.F_3_, the seven genotype combinations remained the same post FBZ treatment. However, there was a significant shift towards the F200Y homozygous resistant genotype after treatment (Shannon diversity indices chi square probability p < 0.001) with 46.0% of the larvae having SS-RR (codon167-200) genotypes, and a reduction in homozygous SS-SS genotypes (Table 3). Where homozygous resistant genotypes occurred at codon 167, this was observed in combination with SR genotypes at codon 200. MHco18/3.F_3_ had the same number of genotype combinations (n = 7) post FBZ treatment, but there was a shift from 34.6% to 45.7% of the genotypes occurring with the either heterozygous or homozygous resistant F167Y. The homozygous susceptible F200Y genotype was only seen in combination with the homozygous resistant F167Y genotype post FBZ treatment. For the homozygous resistant F167Y genotype, all possible F200Y genotypes were observed at a similar level to parental MHco18, including homozygous resistant genotypes, i.e. RR-RR, at both SNPs (3.7%).

**Table 3:** Genotype frequency with percentages shown in parenthesis of the nine possible F167Y/F200Y genotype combinations observed for the parental isolates and genetic cross generations using pyrosequencing on individual larvae; where F_3_ generations have results for untreated and post fenbendazole (BZ) drug treatment.

Genotype		MHco3 (ISE)	MHco18 (UGA2004)	MHco3/18.F_1_	MHco3/18.F_2_	MHco3/18.F_3_	MHco3/18.F_3_.BZ	MHco18/3.F_1_	MHco18/3.F_2_	MHco18/3.F_3_	MHco18/3.F_3_.BZ
SS-SS	TT-TT	84 (100.0)	0 (0.0)	0 (0.0)	23 (33.3)	25 (32.5)	2 (2.6)	0 (0.0)	17 (21.3)	17 (30.9)	0 (0.0)
SS-SR	TT-AT	0 (0.0)	0 (0.0)	66 (81.5)	25 (36.2)	26 (33.8)	22 (29.0)	56 (63.6)	33 (41.3)	12 (21.8)	15 (18.5)
SS-RR	TT-AA	0 (0.0)	36 (42.4)	0 (0.0)	8 (11.6)	9 (11.7)	35 (46.1)	0 (0.0)	5 (6.3)	7 (12.7)	29 (35.8)
RS-SS	AT-TT	0 (0.0)	0 (0.0)	3 (3.7)	1 (1.5)	4 (5.2)	2 (2.7)	10 (11.4)	3 (3.8)	6 (10.9)	0 (0.0)
RS-RS	AT-AT	0 (0.0)	15 (17.7)	12 (14.8)	7 (10.1)	11 (14.3)	8 (10.5)	22 (25.0)	8 (10.0)	10 (18.2)	14 (17.3)
RS-RR	AT-AA	0 (0.0)	28 (33.0)	0 (0.0)	5 (7.3)	1 (1.3)	6 (7.9)	0 (0.0)	13 (16.3)	2 (3.6)	14 (17.3)
RR-SS	AA-TT	0 (0.0)	2 (2.4)	0 (0.0)	0 (0.0)	0 (0.0)	0 (0.0)	0 (0.0)	0 (0.0)	1 (1.8)	2 (2.5)
RR-RS	AA-AT	0 (0.0)	1 (1.2)	0 (0.0)	0 (0.0)	1 (1.3)	1 (1.3)	0 (0.0)	1 (1.3)	0 (0.0)	4 (4.9)
RR-RR	AA-AA	0 (0.0)	3 (3.5)	0 (0.0)	0 (0.0)	0 (0.0)	0 (0.0)	0 (0.0)	0 (0.0)	0 (0.0)	3 (3.7)

#### 4.4.2 Hardy Weinberg Equilibrium and Fixation index

The individual genotype frequencies for both F167Y and F200Y obtained from each of the filial generations and BZ treated F_3_ populations by pyrosequencing were analysed using a chi square test for agreement with the Hardy Weinberg Equilibrium (HWE). The F_1_ generations of the genetic crosses had higher than expected frequencies of heterozygote genotypes at both SNPs. MHco3/18.F_1_ observed heterozygote frequency of 15 (13.6 expected according to HWE) at codon 167 was not significant but at codon 200 was highly significant (χ^2^ = 66.47, p < 0.001) with 77 observed heterozygotes (40 expected). MHco18/3.F_1_ had a significant number of heterozygotes at both SNPs with 32 observed at codon 167 (26.18 expected; χ^2^ = 4.34, p < 0.05) and 78 heterozygotes observed at codon 200 (43.43 expected; χ^2^ = 55.75, p < 0.001). These were the only genotypes from all that were analysed by pyrosequencing of individuals to show any significance in the chi square test that deviate from the HWE. The F_1_ results would corroborate with the Fixation Index ‘F’ value being close to −1 (where negative values indicate heterozygosity) for both F_1_ crosses (MHco3/18.F_1_ F = −0.906, MHco18/3.F_1_ F = −0.796) at the F200Y loci. It also supports the success of both genetic crosses, given the predominant genotypes are heterozygous.

#### 4.4.3 Allele frequency

**Fig. 5:**
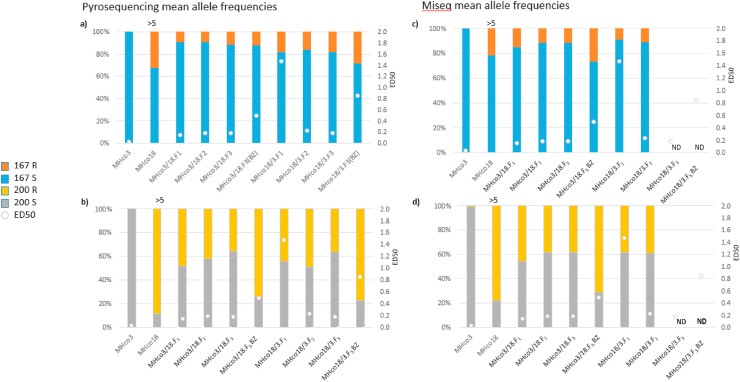
Mean Allele frequencies split over 4 panels a–d with ED50 estimates displayed in white dots with the exception of MHco18 where it is displayed above the column in text. a. Shows the mean allele frequencies of F167Y using pyrosequencing on individual larvae. b. Shows the mean allele frequencies of F200Y using pyrosequencing on individual larvae. c. Shows the mean allele frequencies of F167Y using Miseq on pools of larvae where ND = no data. d. Shows the mean allele frequencies of F200Y using Miseq on pools of larvae where ND = no data.

The pyrosequencing and MiSeq results showed MHco3(ISE) had almost 100% susceptible alleles for the F167Y and F200Y SNPs (1.0% F200Y R with MiSeq). MHco18(UGA2004) had the highest resistant allele frequency at 88.2% and 77.8% for F200Y resistant (R), whereas the F167Y R allele frequency was lower at 32.4% and 21.6% with pyrosequencing and MiSeq, respectively (Fig. 5a–d). MHco3/18 F167Y R allele showed a slight decrease from F_1_ but remained consistent across all the other filial generations. For the F200Y R allele, MHco3/18 has a similar pattern to F167Y with a slight decrease from F_1_, however, after FBZ treatment there was almost a two-fold increase seen in the resistance allele. The MHco18/3 F167Y R allele levels were inconsistent between the methods as pyrosequencing individuals showed a slight decrease between F_1_ and F_2_ generations (18.2 and 16.3% respectively) and MiSeq on pools showed a slight increase for F167Y R between F_1_ and F_2_ (9.2 and 11.3%, respectively). For the F200Y R allele, both methods were in agreement with a slight increase between F_1_ and F_2_ generations. MHco18/3.F_3_ was only analysed by pyrosequencing and showed a slight decline in the R allele at codon 200 between F_2_–F_3_ (48.7 and 38.4%, respectively), however, this increased over two-fold post FBZ treatment (Fig. 5b). An increase of 1.5-fold was also seen at the F167Y R allele post-FBZ treatment in MHco18/3.F_3_ (Fig. 5a). MiSeq data observed the F200Y R and F167Y R double mutants were present at 0.1% in MHco18(UGA2004). For MHco3/18.F_1_, MHco3/18.F_2_, MHco3/18.F_3,_ and MHco3/18.F_3_ BZ crosses, the F200Y R and F167Y R double mutants were present at 2.2%, 0.7%, 1.6% and 0.03%, respectively. For MHco18/3.F_1_, and MHco18/3.F_2_ crosses, the F200Y R and F167Y R double mutants were present at 1.3% and 0.2%, respectively (data not shown). The MiSeq data also confirmed that no changes were observed at codon 198 in either of the parents or genetic cross populations (Doyle et al., 2021).

Overall, the allele frequency at both SNPs using deep amplicon sequencing on pools and pyrosequencing individual larvae from the parents and each filial generation of the crosses produced similar results (Fig. 5). To test the concordance of the two genotyping approaches, we compared the allele frequency from each using the Lin's Concordance Correlation Coefficient (CCC). The results from each method were comparable, with the CCC showing good correlation for F200Y (*CCC* = 0.97; with 95% CI = 0.89–0.99) and moderately correlated for F167Y (*CCC* = 0.55; with 95% CI = −0.17-0.89).

Pairwise calculations of Shannon's Diversity Indices using pyrosequencing data and subsequent G-tests on the two loci using GenAlEx showed that all genetic cross populations, with the exception of one, were significantly different from the parents at both loci (p < 0.01) (see Supplementary Table S2). MHco18/3.F_3_.BZ showed no significant difference at F167Y compared to the MHco18(UGA2004) parent. The F200Y allele frequencies observed in the three filial generations of both genetic crosses were significantly different from both the F_3_.BZ post-treatment populations (p < 0.001). The F200Y allele frequency was also significantly different between the MHco18/3.F_2_ and both F_3_ untreated genetic cross generations (p < 0.05). For F167Y, the most significant differences were observed between the MHco3/18.F_1_, F_2_ and F_3_ (including F_3_.BZ) against MHco18/3.F_3_.BZ (p < 0.001). Significant differences (p < 0.001) were seen between MHco18/3.F_2_ and MHco18/3.F_3_.BZ. There were also significant differences between the F_1_ genetic crosses, including MHco18.3.F_1_ compared to MHco3/18.F_2_ and MHco18/3.F_3_.BZ and MHco18/3.F_3_ compared to MHco18/3.F_3_.BZ at codon 167 (p < 0.05) (See Supplementary Table S2).

## 5 Discussion

The study used classical reciprocal genetic crossing techniques combined with in-depth phenotypic and genotypic analyses to explore the inheritance of BZ resistance and two of the commonly associated resistance SNPs in the β-tubulin isotype 1 gene. A key finding of this work was that, unexpectedly, phenotypic differences in the levels of BZ resistance between the reciprocal crosses were observed. Both the overall total egg output and ED_50_ estimates of the MHco18/3 genetic cross filial progeny were 2.0- and 2.6-fold higher, respectively, than those of the corresponding MHco3/18 genetic cross filial progeny. With the exception of the BZ selected F_3_ populations, these phenotypic differences were not associated with genotypic variation at the β-tubulin isotype 1 locus. This implies differences in the phenotypic outcome was dependent on whether the resistant allele was maternally or paternally inherited.

The reciprocal genetic crosses using the susceptible MHco3(ISE) and BZ-resistant MHco18(UGA2004) *H. contortus* isolates were validated using microsatellite markers. Microsatellite analysis of the parent isolates was shown to cluster into two distinct populations with an F_ST_ value indicating high genetic differentiation (Wright, 1978; Hendrick, 2000; Peakall and Smouse, 2012). Analysis of the F_1_ genetic crosses demonstrates that the alleles have been successfully admixed from MHco3(ISE) and MHco18(UGA2004) *H. contortus* isolates. Both F_1_ genetic crosses show clustering with alleles observed in the parental isolates with F_ST_ reduced to around 0.08 or less. Importantly, when individual worms of F_1_ genetic crosses were genotyped with the panel of six microsatellite markers, their genetic background was intermediate of the MHco3(ISE) and MHco18(UGA2004) *H. contortus* isolates.

Analysis of the egg hatch data showed that both F_1_ generations had ED_50_ estimates higher than the susceptible isolate but lower than the resistant isolate which was in agreement with previous studies looking into the inheritance of BZ resistance using reciprocal crosses with *H. contortus* and *T. colubriformis* (Le Jambre et al., 1979; Herlich et al., 1981; Martin et al., 1988; Sangster et al., 1998; Hunt et al., 2010). In *Haemonchus,* different genetic mechanisms of BZ resistance have been reported, with some suggesting that BZ resistance is semi-dominant (Le Jambre et al., 1979), whilst others have suggested that it is a fully recessive trait (Herlich et al., 1981). Degrees of dominance estimates using Falconer's equation from the two reciprocal crosses suggested incomplete recessivity in one cross and incomplete dominance in the other. The findings illustrate the complexity involved in investigating anthelmintic resistance and that inherent inter- or intra-isolate differences may both play a role in the phenotypic expression of BZ resistance. Work on *Caenorhabditis elegans* has reported that genetically identical individuals can have differing phenotypic responses potentially due to heterogeneity in gene expression (Viney and Diaz, 2012). Additionally the process by which resistance is selected (e.g under or suboptimal dosing compared to frequent dosing) may influence the phenotypic outcome (Sargison, 2016). Studies on *H. contortus* from farms in USA found that the L198V variant of isotype 2 correlated to higher EC_50_ estimates of benzimidazole resistance than that conferred by the F200Y variant alone (Doyle et al., 2021).

The individual and cummaltive FWECs/outputs were significantly higher in the MHco18/3 isolate compared to MHco3/18 at all stages of selection, albeit with reduced impact at each subsequent generation. The findings highlight that phenotype and factors such as parasite fitness and plasticity may be interlinked. A similar finding with increased faecal worm egg outputs and reduced time to patency was observed in *T. circumcincta* isolates that were selected for monepantel resistance (Bartley et al., 2015). In general, the MHco18/3 genetic cross filial generations had higher ED_50_ estimates compared to the MHco3/18 filial generations, indicating greater phenotypic resistance *in vitro*. The nine-fold difference observed in ED_50_ estimates between the F_1_ generations with BZ resistant (MHco18/3) or susceptible (MHco3/18) female parents suggested that there was some positive influence on the resistance phenotype coming from the dam of the cross. The topic of matroclinous influence on *in vitro* expression of BZ resistance has been previously investigated using genetic cross studies, with equivocal findings. Sangster et al. (1998) reported little to no influence; the larval development test used in their study showed that the LC_50_ estimates of both of the F_1_ generations of the reciprocal genetic crosses were around three times greater than those of the susceptible parental isolate and it was noted that this difference was lost in the F_2_ generations. On the other hand, Le Jambre et al. (1979) reported that the progeny of resistant females crossed with non-resistant males had 2.2x higher EHT ED_50_ estimates than the progeny of the reciprocal cross, and that this was maintained through subsequent generations. These results led Le Jambre and colleagues to suggest in 1979 that there was an element of cytoplasmic/extra nuclear factor inheritance, also known as cytoplasmic inheritance, involved in BZ resistance rather than solely the traditional nuclear inheritance. A maternal or cytoplasmic effect has been proposed as a mechanism for inheritance of resistance to the macrocyclic lactone anthelmintic, abamectin, in the carmine spider mite *Tetranychus cinnabarinus* (He et al., 2009), but studies looking at macrocyclic lactone resistance in nematodes found no non-chromosomal influence (Le Jambre et al., 1999). Differences in phenotypic responses have also been observed at different developmental stages (Le Jambre et al., 1979; Kotze 1997), suggesting that the importance of different mechanisms may be at play throughout the life of the nematode, the crosses were undertaken through the transfer of juvenile worms whereas the egg hatch test is looking at egg to L_1_ development. The involvement of other non-specific mechanisms in the expression of BZ resistance has been proposed, including ABC transporters (Kerboeuf et al., 1999), cytochrome P450 enzymes (reviewed by Matouskova et al., 2016), and microRNAs (Devaney et al., 2010).

Assessment of the β-tubulin associated SNPs of each of the filial generations provided interesting findings. The F_1_ progeny of both reciprocal crosses show the expected genotypes associated with successful crossing, but these do not account for the greatly different EHT ED_50_ phenotype results. Genotyping individuals from each population offered an advantage over sequencing of pooled populations of being able to report actual genotypes and resultant combinations for each SNP. Consequently this allows us to present the first case of a double homozygous resistant genotype at both codons 167 and 200. This genotype combination was found in both the MHco18(UGA2004) parent L_3_ and in the MHco18/3.F_3_.BZ L_3_ population. The MiSeq assay results also intimated that this combination was also observed in pools of larvae. This has not been found previously despite the large number of genotyping studies conducted on Trichostrongylid nematodes (Mottier and Prichard, 2008; Hodgkinson et al., 2008; Barrere et al., 2012; Kotze et al., 2012; Redman et al., 2015; Atanásio-Nhacumbe et al., 2019); it has been considered that having a combination of two homozygous resistant genotypes in the β-tubulin isotype 1 gene would be lethal (Mottier and Prichard, 2008) and that even in the heterozyote, the resistant alleles are never on the same strand, i.e. the variants are always in trans, not in cis. In this study, these double homozygous resistant genotypes have only been reported in the L_3_ stage of the parasite and it has never been looked at in adults to see if these individuals can undergo normal development in the host and be sexually reproductive. The fact they have hatched from eggs and developed to L_3_ shows that the mutation is not lethal to this stage of development.

It has been reported by Barrere et al. (2012) that a heterozygous genotype at both F167Y and F200Y confers a resistant phenotype, capable of surviving three times the recommended dose rate of albendazole. In this study, the main difference between the two crosses in the F_2_ and F_3_ generations was that the MHco18/3 had higher resistance allele frequencies at F167Y, which was most noticeable after BZ treatment of the F_3_ population. In the resistant parent isolate 57.6% of the genotypes were either heterozygous or homozygous resistant at codon 167. Perhaps the F167Y in the MHco18(UGA2004) parental isolate is important in conferring a resistant phenotype. MHco18/3.F_1_ had 25.0% SR-SR genotype, whereas MHco3/18.F_1_ had 14.8%. The other genotype combinations for the F_1_ populations were heterozygous at either SNP position, potentially conferring a more susceptible phenotype. The other filial generations had similar levels of double heterozygotes to each other.

Previous work carried out by Sargison et al. (2019a) investigating mating barriers between different *H. contortus* isolates suggested that MHco3(ISE) females were more likely to produce progeny from matings with their own isolate when co-infected with two other genetically/geographically different isolates. However, it was noted that females from the two other *H. contortus* isolates did not show this attribute towards MHco3(ISE), and freely mated between the two isolates with the co-infections being tested. This study design did not allow for isolate choices in mating, but it is possible that sub-populations with particular morphological features influenced the mating behaviour, resulting in an apparent sex-linked difference that manifested in the resistance phenotype. It was not possible to investigate this within the present study. As mentioned above, the possibility of other non-specific or extra-nuclear mechanisms of resistance being involved can not be precluded and requires further investigation.

### 5.1 Conclusions

This is the first trichostrongyle gastrointestinal nematode genetic crossing study where individual genotypes at the β-tubulin isotype 1 gene were investigated alongside phenotypic indices. The apparent less than perfect correlation between phenotype and genotype demonstrated that their relationship is complex and that multiple genes/mechanisms may be involved in BZ resistance and that β-tubulin only explains part of the phenotypic variance, and/or that the phenotypic tools used for assessing ED_50_ are too insensitive to correlate with what is observed genotypically. This study has confirmed previous studies’ findings in that the inheritance of BZ resistance is influenced by maternal and/or cytoplasmic mechanisms. The work has for the first time demonstrated that, albeit extremely rare, double homozygous resistant genotypes at positions 167 and 200 on the β-tubulin isotype 1 gene are viable and do not preclude the development from egg to infective larvae stage and further work investigating the potential for the further development of these individual L_3_ to progress to fertile adults is required to assess whether it is a unique characteristic for the MHco18(UGA2004) isolate.

## Declaration of competing interest

The authors declare no conflict of interest.
