# Supplementary material for: Phenotypic and genotypic analysis of benzimidazole resistance in reciprocal genetic crosses of Haemonchus contortus
Source: Int J Parasitol Drugs Drug Resist. 2021 Dec 1;18:1–11. doi: 10.1016/j.ijpddr.2021.11.001 (PMC8666523; doi:10.1016/j.ijpddr.2021.11.001)
Supplement: Multimedia component 1 [file mmc1.docx]

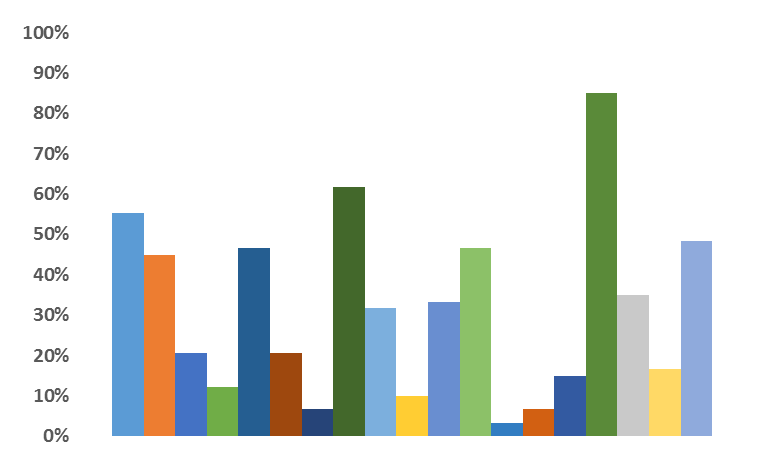


**Frequency**

**204**

**218**

**Hcms33**

**192**

**196**

**232**

**240**

**Hcms8a20**

**196**

**202**

**205**

**Hcms53265**

**203**

**205**

**207**

**209**

**211**

**Hcms25**

**234**

**250**

**Hcms22c03**

**213**

**215**

**221**

**Hcms22193**


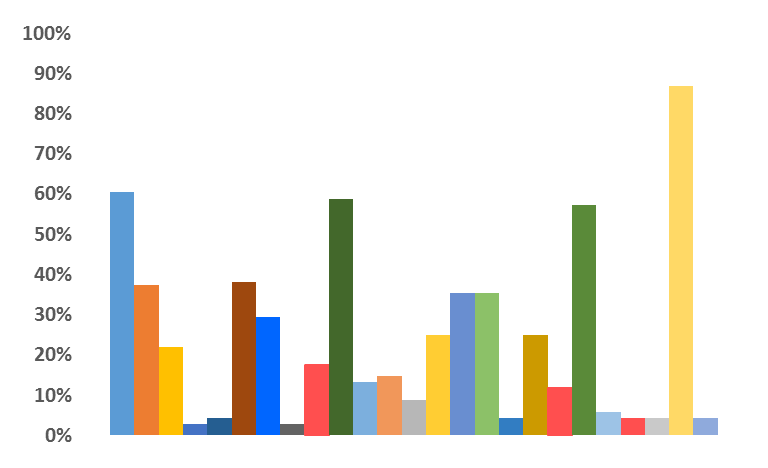


**Frequency**

**Alleles**

**204**

**218**

**Hcms33**

**200**

**220**

**232**

**240**

**248**

**252**

**Hcms8a20**

**199**

**202**

**205**

**211**

**214**

**Hcms53265**

**203**

**205**

**207**

**209**

**Hcms25**

**242**

**246**

**250**

**258**

**Hcms22c03**

**211**

**213**

**215**

**221**

**Hcms22193**

**Alleles**

**a**

**b**
