# Supplementary material for: Phenotypic and genotypic analysis of benzimidazole resistance in reciprocal genetic crosses of Haemonchus contortus
Source: Int J Parasitol Drugs Drug Resist. 2021 Dec 1;18:1–11. doi: 10.1016/j.ijpddr.2021.11.001 (PMC8666523; doi:10.1016/j.ijpddr.2021.11.001)
Supplement: Multimedia component 2 [file mmc2.docx]

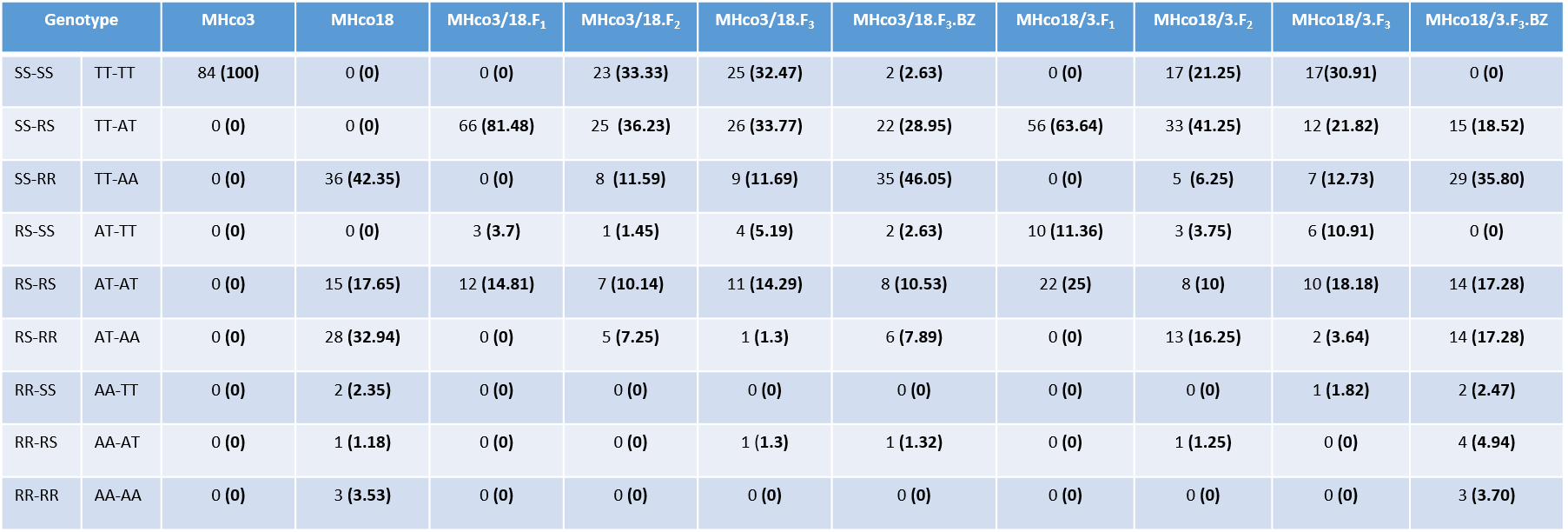


Table 3. Genotype frequency with percentages shown in bold, in brackets of the nine possible F167Y / F200Y genotype combinations observed for the parental and genetic cross generations using pyrosequencing on individual larvae; where F_3_ generations have results for untreated and post FBZ drug treatment.


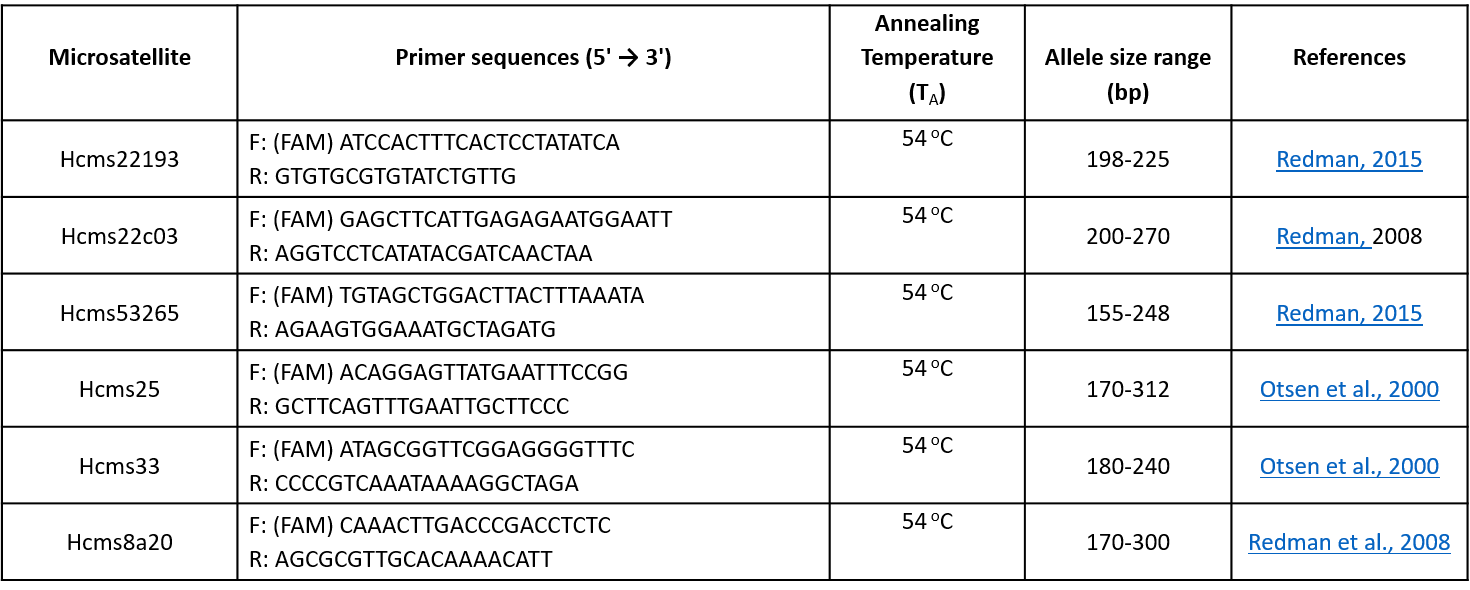


**Supplementary Table S1:** Panel of microsatellites used for population genetics analysis of *H. contortus*
