# Supplementary material for: Phenotypic and genotypic analysis of benzimidazole resistance in reciprocal genetic crosses of Haemonchus contortus
Source: Int J Parasitol Drugs Drug Resist. 2021 Dec 1;18:1–11. doi: 10.1016/j.ijpddr.2021.11.001 (PMC8666523; doi:10.1016/j.ijpddr.2021.11.001)
Supplement: Multimedia component 3 [file mmc3.docx]

Supplementary Table S2 Showing summary of Shannon Analysis over Loci for all Pairwise Population Combinations parental isolates and filial generations of genetic crosses

where ns=not significant, * P<0.05, ** P<0.01, *** P<0.001

| Comparison of Populations | |  |  | F167Y |  |  |  |  | F200Y |  |  |
| --- | --- | --- | --- | --- | --- | --- | --- | --- | --- | --- | --- |
| **Vs** | | **No alleles Pop1** | **No alleles Pop2** | **G** | **Chi Prob** | **Chi Signif** | **No alleles Pop1** | **No alleles Pop2** | **G** | **Chi Prob** | **Chi Signif** |
| MHco18 | MHco3/18.F1 | 170 | 162 | 26.869 | 0.000 | *** | 170 | 162 | 67.015 | 0.000 | *** |
| MHco18 | MHco18/3.F1 | 170 | 176 | 8.608 | 0.003 | ** | 170 | 176 | 79.211 | 0.000 | *** |
| MHco18 | MHco3/18.F2 | 170 | 138 | 23.957 | 0.000 | *** | 170 | 138 | 77.354 | 0.000 | *** |
| MHco18 | MHco18/3.F2 | 170 | 160 | 11.005 | 0.001 | *** | 170 | 160 | 63.266 | 0.000 | *** |
| MHco18 | MHco3/18.F3 | 170 | 156 | 20.143 | 0.000 | *** | 170 | 154 | 95.506 | 0.000 | *** |
| MHco18 | MHco3/18.F3_BZ | 170 | 152 | 17.646 | 0.000 | *** | 170 | 152 | 10.445 | 0.001 | ** |
| MHco18 | MHco18/3.F3 | 170 | 110 | 7.561 | 0.006 | ** | 170 | 110 | 84.290 | 0.000 | *** |
| MHco18 | MHco18/3.F3_BZ | 170 | 162 | 0.448 | 0.503 | ns | 170 | 162 | 7.231 | 0.007 | ** |
| MHco3 | MHco18 | 168 | 170 | 84.423 | 0.000 | *** | 168 | 170 | 341.135 | 0.000 | *** |
| MHco3 | MHco3/18.F1 | 168 | 162 | 22.086 | 0.000 | *** | 168 | 162 | 134.376 | 0.000 | *** |
| MHco3 | MHco18/3.F1 | 168 | 176 | 46.023 | 0.000 | *** | 168 | 176 | 126.585 | 0.000 | *** |
| MHco3 | MHco3/18.F2 | 168 | 138 | 21.409 | 0.000 | *** | 168 | 138 | 109.376 | 0.000 | *** |
| MHco3 | MHco18/3.F2 | 168 | 160 | 39.684 | 0.000 | *** | 168 | 160 | 138.133 | 0.000 | *** |
| MHco3 | MHco3/18.F3 | 168 | 156 | 27.455 | 0.000 | *** | 168 | 154 | 99.684 | 0.000 | *** |
| MHco3 | MHco3/18.F3_BZ | 168 | 152 | 29.618 | 0.000 | *** | 168 | 152 | 242.478 | 0.000 | *** |
| MHco3 | MHco18/3.F3 | 168 | 110 | 37.391 | 0.000 | *** | 168 | 110 | 84.840 | 0.000 | *** |
| MHco3 | MHco18/3.F3_BZ | 168 | 162 | 73.235 | 0.000 | *** | 168 | 162 | 263.794 | 0.000 | *** |
| MHco18/3.F1 | MHco3/18.F2 | 176 | 138 | 5.013 | 0.025 | * | 176 | 138 | 0.165 | 0.684 | ns |
| MHco18/3.F1 | MHco18/3.F2 | 176 | 160 | 0.219 | 0.639 | ns | 176 | 160 | 0.662 | 0.416 | ns |
| MHco18/3.F1 | MHco3/18.F3 | 176 | 156 | 2.895 | 0.089 | ns | 176 | 154 | 1.505 | 0.220 | ns |
| MHco18/3.F1 | MHco3/18.F3_BZ | 176 | 152 | 2.030 | 0.154 | ns | 176 | 152 | 30.951 | 0.000 | *** |
| MHco18/3.F1 | MHco18/3.F3 | 176 | 110 | 0.000 | 1.000 | ns | 176 | 110 | 1.778 | 0.182 | ns |
| MHco18/3.F1 | MHco18/3.F3_BZ | 176 | 162 | 4.968 | 0.026 | * | 176 | 162 | 38.988 | 0.000 | *** |
| MHco3/18.F1 | MHco18/3.F1 | 162 | 176 | 5.741 | 0.017 | * | 162 | 176 | 0.351 | 0.554 | ns |
| MHco3/18.F1 | MHco3/18.F2 | 162 | 138 | 0.000 | 1.000 | ns | 162 | 138 | 0.912 | 0.339 | ns |
| MHco3/18.F1 | MHco18/3.F2 | 162 | 160 | 3.577 | 0.059 | ns | 162 | 160 | 0.048 | 0.827 | ns |
| MHco3/18.F1 | MHco3/18.F3 | 162 | 156 | 0.444 | 0.505 | ns | 162 | 154 | 3.149 | 0.076 | ns |
| MHco3/18.F1 | MHco3/18.F3_BZ | 162 | 152 | 0.853 | 0.356 | ns | 162 | 152 | 24.023 | 0.000 | *** |
| MHco3/18.F1 | MHco18/3.F3 | 162 | 110 | 3.770 | 0.052 | ns | 162 | 110 | 3.355 | 0.067 | ns |
| MHco3/18.F1 | MHco18/3.F3_BZ | 162 | 162 | 20.176 | 0.000 | *** | 162 | 162 | 30.921 | 0.000 | *** |
| MHco18/3.F2 | MHco3/18.F3 | 160 | 156 | 1.471 | 0.225 | ns | 160 | 154 | 3.939 | 0.047 | * |
| MHco18/3.F2 | MHco3/18.F3_BZ | 160 | 152 | 0.892 | 0.345 | ns | 160 | 152 | 21.865 | 0.000 | *** |
| MHco18/3.F2 | MHco18/3.F3 | 160 | 110 | 0.049 | 0.825 | ns | 160 | 110 | 4.094 | 0.043 | * |
| MHco18/3.F2 | MHco18/3.F3_BZ | 160 | 162 | 6.914 | 0.009 | ** | 160 | 162 | 28.416 | 0.000 | *** |
| MHco3/18.F2 | MHco18/3.F2 | 138 | 160 | 3.105 | 0.078 | ns | 138 | 160 | 1.351 | 0.245 | ns |
| MHco3/18.F2 | MHco3/18.F3 | 138 | 156 | 0.350 | 0.554 | ns | 138 | 154 | 0.579 | 0.447 | ns |
| MHco3/18.F2 | MHco3/18.F3_BZ | 138 | 152 | 0.704 | 0.402 | ns | 138 | 152 | 31.752 | 0.000 | *** |
| MHco3/18.F2 | MHco18/3.F3 | 138 | 110 | 3.338 | 0.068 | ns | 138 | 110 | 0.824 | 0.364 | ns |
| MHco3/18.F2 | MHco18/3.F3_BZ | 138 | 162 | 17.980 | 0.000 | *** | 138 | 162 | 39.368 | 0.000 | *** |
| MHco18/3.F3 | MHco18/3.F3_BZ | 110 | 162 | 4.587 | 0.032 | * | 110 | 162 | 46.308 | 0.000 | *** |
| MHco3/18.F3 | MHco3/18.F3_BZ | 156 | 152 | 0.067 | 0.795 | ns | 154 | 152 | 42.834 | 0.000 | *** |
| MHco3/18.F3 | MHco18/3.F3 | 156 | 110 | 1.747 | 0.186 | ns | 154 | 110 | 0.046 | 0.829 | ns |
| MHco3/18.F3 | MHco18/3.F3_BZ | 156 | 162 | 14.469 | 0.000 | *** | 154 | 162 | 52.014 | 0.000 | *** |
| MHco3/18.F3_BZ | MHco18/3.F3 | 152 | 110 | 1.160 | 0.281 | ns | 152 | 110 | 38.466 | 0.000 | *** |
| MHco3/18.F3_BZ | MHco18/3.F3_BZ | 152 | 162 | 12.405 | 0.000 | *** | 152 | 162 | 0.339 | 0.560 | ns |
